# AGENESIS OF LUNG - A REPORT OF TWO CASES

**DOI:** 10.4103/0970-2113.44136

**Published:** 2008

**Authors:** KH Kisku, MK Panigrahi, R Sudhakar, A Nagarajan, R Ravikumar, JR Daniel

**Affiliations:** 1 Department of Pulmonary Medicine, Pondicherry Institute of Medical Sciences, Pondicherry - 605014., India; 2 Department of Radiology, Pondicherry Institute of Medical Sciences, Pondicherry - 605014., India

**Keywords:** Recurrent lower respiratory tract infections, Opaque hemithorax, Hypoplasia, Agenesis

## Abstract

Agenesis of lung is a rare congenital disorder. We are reporting varied degree of pulmonary agenesis in two adult patients.

## INTRODUCTION

Agenesis of the lung is an extremely rare congenital anomaly representing failure of development of the primitive lung bud. The condition was first discovered accidentally at the autopsy of an adult female in 1673, by De Pozze^1^. From India, the first case was reported by Muhamed^2^in 1923, of a left sided pulmonary agenesis in a medicolegal autopsy. Munch Meyer^3^diagnosed it clinically in 1885. Subsequently a few more case reports have appeared and by 1977, over 200 cases of under development of the lung have been reported. Most authors describe a single or a small number of cases. The most exhaustive reviews are those of Oyamada et al^4^., Vale^5^, Maltz and Nadas^6^and Sbokos and McMillan^7^.

## CASE REPORT

**Fig. 1a):**
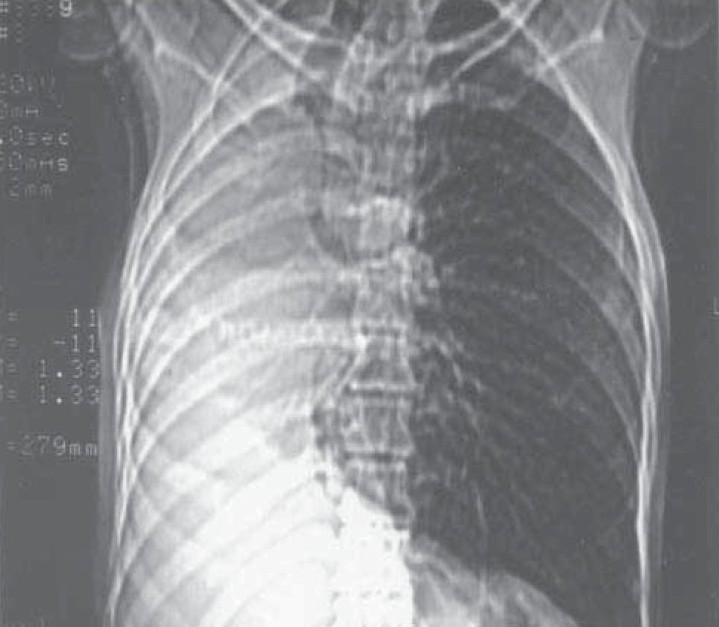
CT Scannogram showing right side opaque hemi thorax with signs of volume loss.

### Case 1

A 35 year old male presented with low grade fever and cough with mucopurulent sputum since 10 days with history of recurrent similar episodes since childhood. General examination revealed pallor and tachypnea. Chest examination showed decreased movement of right hemithorax, ipsilateral tracheal shift, impaired percussion note and absent breath sound on right side with scanty coarse inspiratory crackles in mammary area. The percussion note was hyper resonant on left side along with harsh vesicular breath sound and coarse crackles. Blood routine examination showed mild anemia with neutrophilic leucocytosis. He was otherwise healthy with no other co morbid illness. Chest X ray revealed an opaque right hemi thorax with signs of volume loss and compensatory hyperinflation of the left lung. He was investigated with CT thorax, bronchoscopy, bronchogram, pulmonary angiogram and a diagnosis of Type 2 lung agenesis was made (figure 1.a,b,c,d). No other congenital anomalies were identified.

**Fig. 1b):**
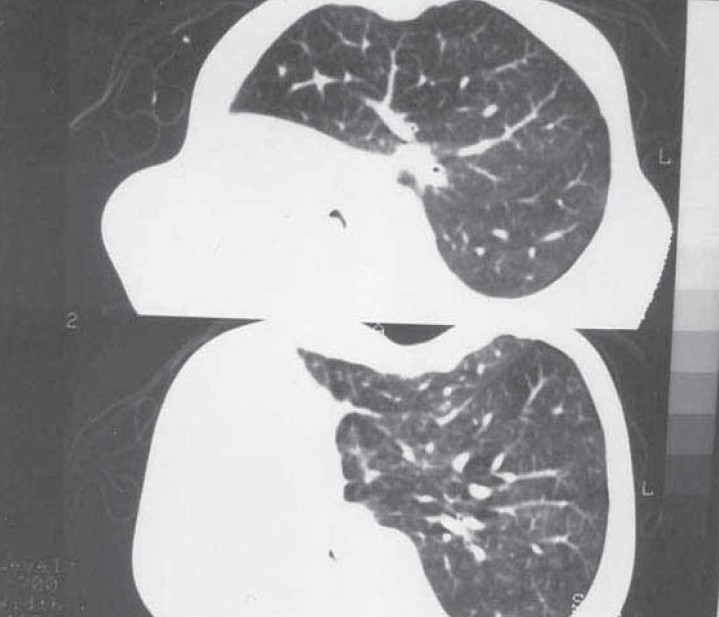
CT chest showing absent aerated lung on the right side with herniation of the left lung.

**Fig. 1c):**
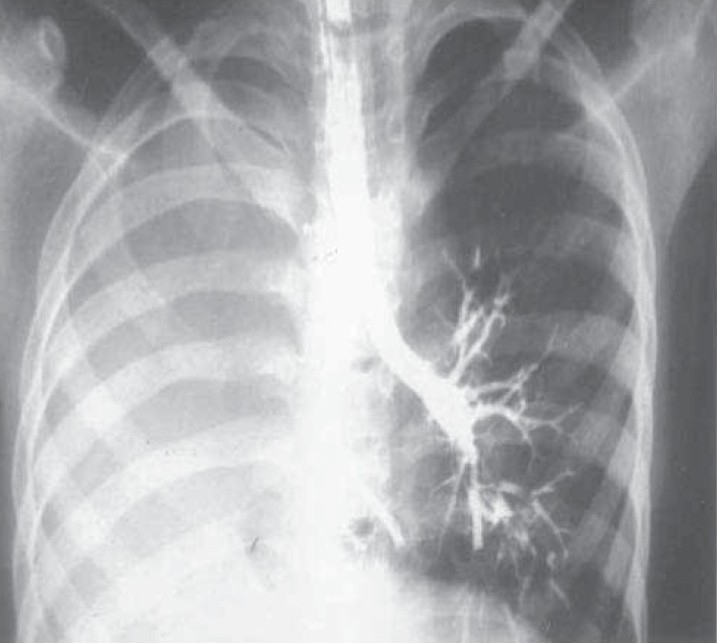
Bronchogram showing right sided rudimentary bronchus.

**Fig. 1d):**
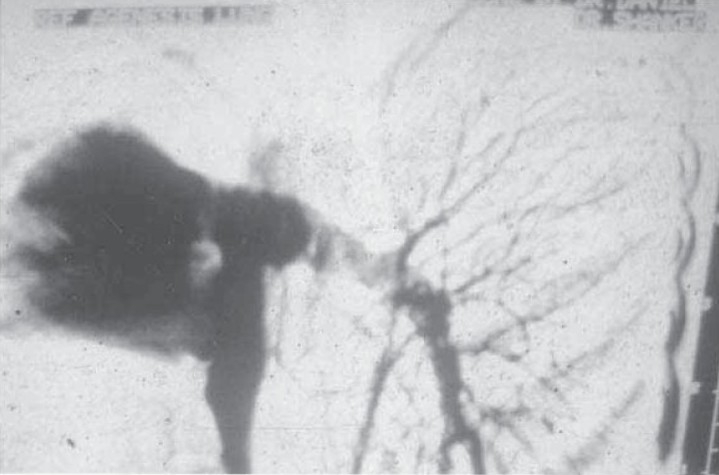
Pulmonary angiogram showing absent right pulmonary artery.

### Case 2

A 39 year old male presented with recurrent cough with expectoration since childhood and grade II dyspnoea with no other comorbid illness. On examination he was of short stature with scoliosis to the left, bronchial breath sound on the left side with few crackles. Routine blood examination, sonography of abdomen and echocardiography of heart were normal. Chest X ray showed left side opaque hemi thorax with right side compensatory hyperinflation. CT chest showed left sided minimal lung shadow with herniation of right lung and a narrowed left pulmonary artery. Bronchoscopy, pulmonary angiogram, bronchogram were done and a diagnosis of Type 3 lung agenesis was made (figure 2.a,b,c,d). No other congenital anomalies were identified.

**Fig. 2a):**
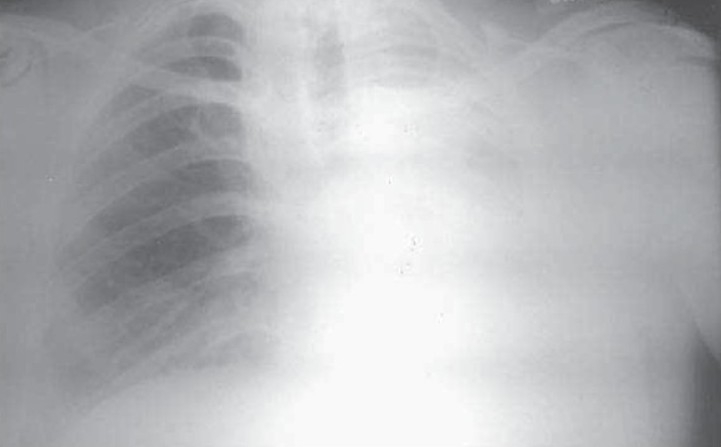
Chest X- ray showing opaque left hemithorax with signs of volume loss.

**Fig. 2b):**
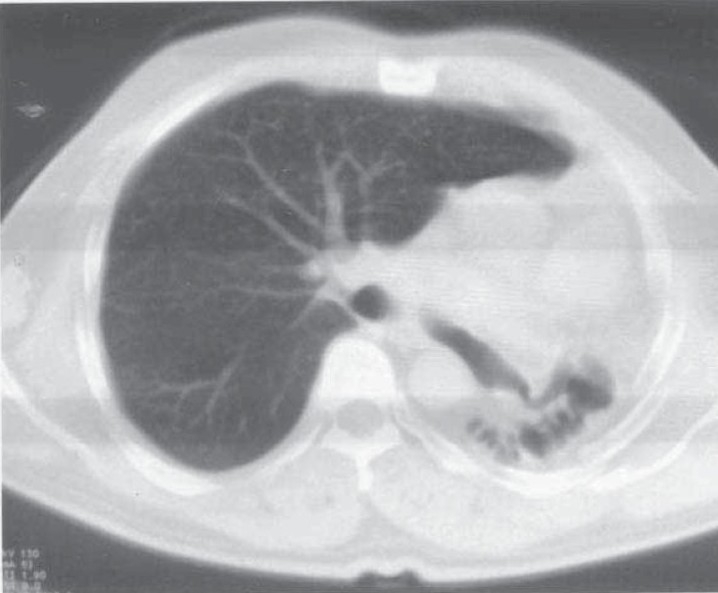
CT chest showing minimal aerated lung on the left side with herniation of the right lung.

**Fig. 2c):**
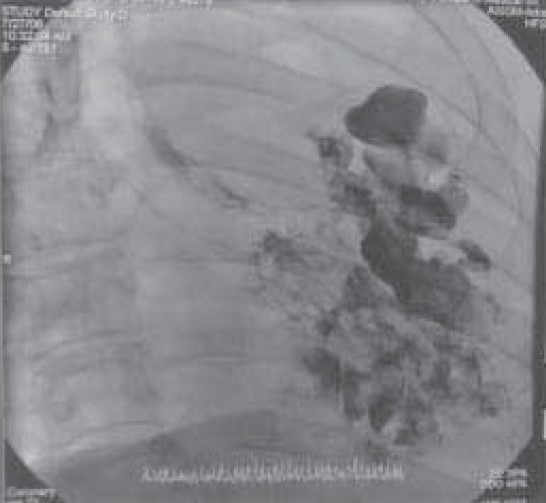
Bronchogram showing reduced air spaces on the left side and a normal left main bronchus. Normal on the right side.

**Fig. 2d):**
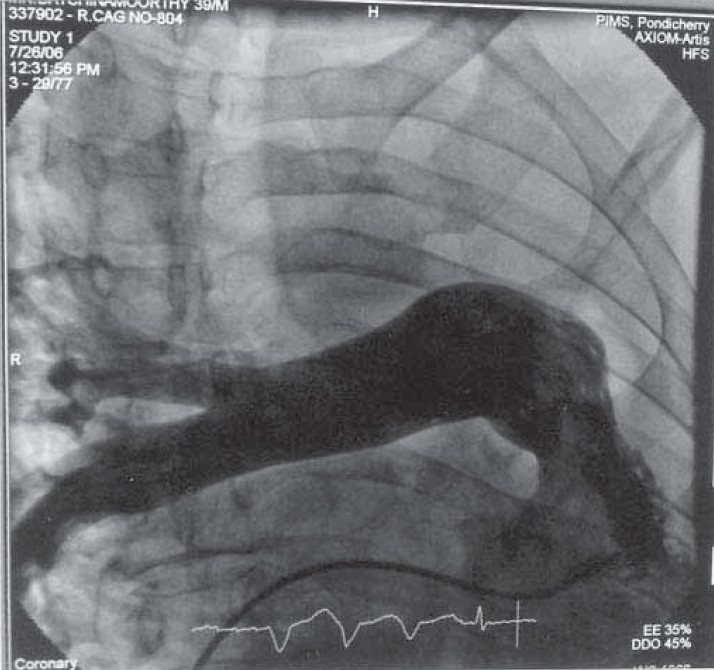
Pulmonary angiogram showing absent pulmonary artery on the left side.

## DISCUSSION

Schneider^8^classified agenesis into three groups, which has been subsequently modified by Boyden^9^. Depending upon the stage of development of the primitive lung bud, pulmonary agenesis is classified into three categories:

Type 1(Agenesis) – Complete absence of lung and bronchus and no vascular supply to the affected side.

Type 2 (Aplasia) – Rudimentary bronchus with complete absence of pulmonary parenchyma.

Type 3 (Hypoplasia) – Presence of variable amounts of bronchial tree, pulmonary parenchyma and supporting vasculature.

Our patients would classify as Type 2 (Case 1) and Type 3 (Case 2) pulmonary agenesis.

The onset of symptoms in pulmonary agenesis is remarkably variable. In many cases, presence of this anomaly usually comes to light during infancy because of recurrent chest infections, cardiopulmonary insufficiency or due to associated congenital anomalies. However, patients with one lung have been reported to survive well into adulthood without much complaints. The oldest patient cited by Oyamada et al,^4^ was 72 years old. The present cases are few of the older cases of aplasia reported in the literature.

Our patients had varied respiratory complaints, one had a brief respiratory infection and the other had recurrent respiratory infections since childhood. Diagnosis was suspected from routine radiological examination of chest, where possibilities of atelectasis or agenesis of the left lung were considered. The diagnosis was confirmed by bronchography and pulmonary angiography.

Nearly 50% cases of pulmonary agenesis have associated congenital defects^7^, involving cardiovascular, skeletal, gastrointestinal and genitourinary system. None of the two patients had associated congenital defect.

The exact aetiology of this condition is unknown although genetic factors, viral agents and dietary deficiency of Vitamin A during pregnancy have been implicated^7^. Left sided agenesis is more common and these subjects have a longer life expectancy than those with right sided agenesis^4^. This is probably due to excessive mediastinal shift and malrotation of carina in right sided agenesis which hinders proper drainage of the functioning lung and increases chances of respiratory infections.

The differential diagnosis of the condition in adults include collapse, thickened pleura, destroyed lung and pneumonectomy. Final diagnosis can be established after bronchoscopy, bronchography but, in some cases, angiography is also needed. Surgery is seldom required for agenesis or aplasia, as it can be managed on conservative lines. The prognosis in these cases depends upon the functional integrity of the remaining lung as well as the presence of associated anomalies.
